# Design and performance of a new setup for spatially resolved transmission X-ray photoelectron spectroscopy at the Swiss Light Source

**DOI:** 10.1107/S1600577519002984

**Published:** 2019-04-02

**Authors:** Kanak Roy, Joerg Raabe, Pascal Schifferle, Simone Finizio, Armin Kleibert, Jeroen A. van Bokhoven, Luca Artiglia

**Affiliations:** a Institute for Chemical and Bioengineering, ETH Zürich, Zürich 8093, Switzerland; b Swiss Light Source, Paul Scherrer Institute, Villigen 5232, Switzerland; c Laboratory for Catalysis and Sustainable Chemistry, Paul Scherrer Institute, Villigen 5232, Switzerland; d Laboratory of Environmental Chemistry, Paul Scherrer Institute, Villigen 5232, Switzerland

**Keywords:** X-ray spectromicroscopy, spatial resolution, X-ray photoelectron spectroscopy, scanning transmission X-ray microscope

## Abstract

The successful design, installation and operation of a high spatial resolution X-ray photoelectron spectrometer at the Swiss Light Source is presented. In this instrument, a Fresnel zone plate is used to focus an X-ray beam onto the sample and an electron analyzer positioned at 45° with respect to the incoming beam direction is used to collect photoelectrons from the backside of the sample. By raster scanning the sample, transmitted current, X-ray absorption and X-ray photoemission maps can be simultaneously acquired. This work demonstrates that chemical information can be extracted with micrometre resolution; the results suggest that a spatial resolution better than 100 nm can be achieved with this approach in future. This kind of photoelectron spectromicroscope will allow *in situ* measurements with high spatial resolution also under ambient pressure conditions (in the millibar range). Element-specific X-ray photoemission maps can be obtained before and while exposing the sample to gas/gas mixtures to show morphological and chemical changes of the surface.

## Abstract 2

The design and development of a photoelectron spectromicroscope working in transmission mode are presented.

## 1. Introduction   

The development of multifunctional nanomaterials and their increasing implications demand exploration of nanoscale objects at an unprecedented resolution. A number of scanning probe microscopic (SPM) techniques such as scanning tunneling microscopy (Binnig *et al.*, 1982 ▸) and atomic force microscopy (Binnig *et al.*, 1986 ▸), and electron microscopic techniques (Ross, 2007 ▸) such as high-resolution transmission electron microscopy have developed our understanding at the nanometre to sub-nanometre and even at atomic resolution. SPM techniques allow high spatial resolution to be achieved, but at the same time impose some sample limitations, for example, making the analysis of powders difficult or even impossible. SPM techniques access the morphological information of samples, whereas electron microscopy allows also the chemical characterization of samples through energy-dispersive X-ray microanalysis, although the energy resolution is poor. Spectroscopic findings are often important for a clear understanding of structure–performance relationships. Parallel to microscopic techniques, high-resolution X-ray spectromicroscopic techniques have also been developed. X-ray spectromicroscopy (Hitchcock, 2012 ▸; Günther, 2002 ▸; Kirz & Jacobsen, 2009 ▸; Tonner & Harp, 1988 ▸), which combines X-ray spectroscopy and X-ray imaging, has grown as a powerful method for understanding the chemical speciation and electronic structure with micrometre to nanometre precision. The high spatial resolution of X-ray microscopes, down to a few nanometres, is commonly achieved by using a micro- or nano-probe, *i.e.* illuminating the sample with a focused photon beam. By scanning the probe or the sample, a two-dimensional image of the sample can be obtained. Such maps bear information tailored from X-ray absorption, in the case of scanning transmission X-ray microscopy (STXM), or X-ray photoemission, in the case of scanning methods like scanning photoelectron microscopy (SPEM), or full field methods such as X-ray photoemission electron microscopy (XPEEM). Today these instruments are available in many synchrotron facilities (Kilcoyne *et al.*, 2003 ▸; Wiesemann *et al.*, 2000 ▸; Beetz *et al.*, 2003 ▸; Kaznatcheev *et al.*, 2007 ▸; Shin & Lee, 2001 ▸; Kenney *et al.*, 1989 ▸; Guttmann *et al.*, 2001 ▸; Xue *et al.*, 2010 ▸; Raabe *et al.*, 2008 ▸; Takeichi *et al.*, 2016 ▸; Kaulich *et al.*, 2011 ▸; Dudin *et al.*, 2010 ▸; Bostwick *et al.*, 2012 ▸; Amati *et al.*, 2018 ▸; Kolmakov *et al.*, 2016 ▸; Fraile Rodríguez *et al.*, 2007 ▸) and provide important micro- and nano-analysis of materials and condensed matter.

A crucial parameter of scanning X-ray spectromicroscopy is the illuminating X-ray spot size, which determines the achievable spatial resolution. In high-brilliance synchrotron beamlines, X-ray focusing elements comprising of Fresnel zone plates (FZPs) have become popular to achieve small spot sizes at the cost of some loss of the X-ray flux. We made use of FZP-assisted X-ray focusing optics and a photoelectron detector for our photoelectron spectroscopy setup to achieve a sub-micrometre lateral/spatial resolution in X-ray photoelectron spectroscopy (XPS). We report the design, sample manipulation and successful operation of the setup at the Surface/Interface Microscopy (SIM) beamline of the Swiss Light Source (SLS). Our instrument adopts a transmission geometry whereby the photon source and the electron detector are positioned at opposite sides of the sample. XPS in transmission geometry was also reported by Hovland (1977 ▸) in a scanning Electron Spectroscopy for Chemical Analysis (ESCA) instrument, achieving resolutions better than 20 µm (Hovland, 1977 ▸). The experiments consisted of creating a spatially localized Al *K*α source by bombarding a thin aluminium foil with a focused scanning electron beam. The localized Al *K*α radiation probed a thin sample mounted on the opposite side of the aluminium foil. Such a setup of earlier days had severe limitations due to changes in the background sensitivity and the dispersion of the X-rays in the foil. Scanning probe X-ray techniques today use X-ray focusing systems to create sub-micrometre- or nanometre-probes. The instrument presented in this manuscript achieves a spatial resolution better than 500 nm on a standardized sample and around 1 µm during the proof-of-principle test by using X-ray focusing elements. The focused spot size can be calculated by taking into account the type of zone plate used in the experiment, the beam dimension at the source point (PEEM endstation at the SIM beamline) of 30 µm (horizontal) × 100 µm (vertical), an estimated spectral resolution of 5000, and the distance between the PEEM focal spot and our endstation (4 m). The result is 220 nm × 720 nm; however, we are confident that the resolution can be improved to less than 100 nm by further optimizing the alignment and by using a focused secondary photon source (pinhole) to illuminate the zone plate.

Transmission measurements require thin samples and commonly probe the sample volume, as in the case of STXM. In most of the electron spectromicroscopes the photon source and the electron spectrometer are on the same side of the sample to allow the analysis of thick samples and an easier thermal treatment. However, the availability of customized thin and photon-transparent silicon nitride membranes, and the possibility to create well characterized thin samples directly on them through nanofabrication, facilitate the production of samples suitable for transmission SPEM. The main advantage of a transmission measurement geometry is to bring the focusing unit [order-sorting aperture (OSA) and zone plate] closer to the sample for a better lateral resolution without blocking photoelectrons from reaching the analyzer. By adopting such geometry, *in situ* space-resolved photoemission experiments can be carried out with samples exposed to specific gas/gas mixtures in a wide pressure range. Ambient pressure X-ray photoelectron spectroscopy (APXPS) is a well consolidated tool to investigate the surface of samples up to 100 mbar (Lena *et al.*, 2017 ▸). This is possible thanks to differentially pumped photoelectron analyzers, which are separated from the analysis cell by nozzles, allowing the successful operation of detectors in vacuum, and pre-lenses, which extract and focus photoelectrons generated in the experimental cell (Karslıoğlu & Bluhm, 2017 ▸). One of the current challenges in APXPS is the development of imaging (Amati *et al.*, 2018 ▸; Kolmakov *et al.*, 2016 ▸). Using specific settings of the lenses, new-generation spectrometers can achieve a resolution of a few micrometres. Nowadays, by means of differential pumping and efficient flow and pressure control of the measurement cell, PEEM setups also offer the possibility to carry out measurements up to 1 mbar with an estimated lateral resolution of less than 30 nm when using UV light or electrons for imaging (http://www.specs.de/cms/front_content.php?idcat=382). The main aim of this work is to show an alternative measurement concept and geometry based on transmission SPEM, which can be coupled to any differentially pumped photoelectron analyzer to obtain an *in situ* space-resolved analysis of samples exposed to millibar pressures of gas in the sub-micrometre range. Another strategy to measure photoelectron spectroscopy in the bar range is to use electron semi-transparent membranes to build micro-reaction cells. Graphene-membranes covered *in situ* cells have already demonstrated successful photoemission experiments at atmospheric pressure with gases and liquids (Kolmakov *et al.*, 2011 ▸; Weatherup *et al.*, 2016 ▸; Velasco-Vélez *et al.*, 2016 ▸), and will disseminate as a viable solution for more *operando* studies in future work (Roy *et al.*, 2018 ▸). Devices based on graphene-sealed cells have allowed studies with the conventional SPEM technique *in situ* and under *operando* conditions with *ca*. 100 nm lateral resolution (Al-Hada *et al.*, 2018 ▸), and new-generation TEM flow devices allow a fluid to flow in a 200 nm-thick cell (http://denssolutions.com/products/ocean/nano-cell/). Such cells may also be integrated in this setup, although transmission measurements through micrometres of fluids may be challenging due to X-ray attenuation.

**Figure 1:**
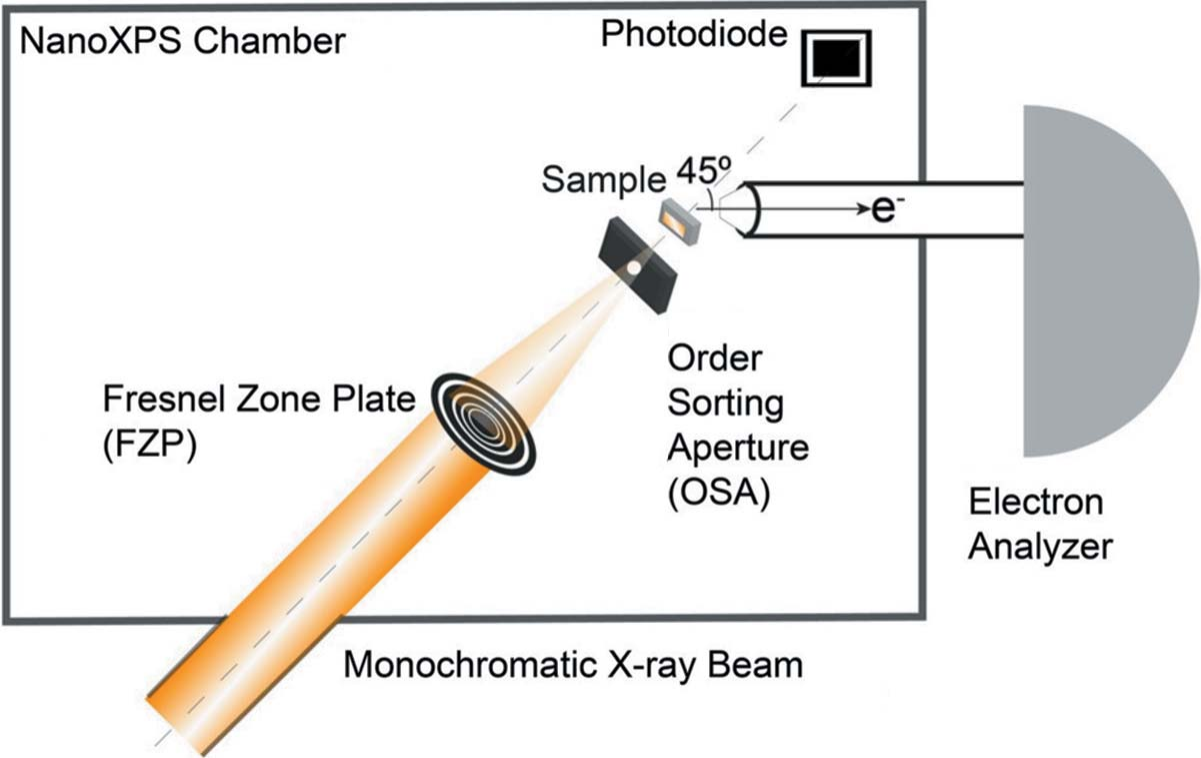
Schematics of the setup displaying all essential components: FZP and OSA used as X-ray focusing elements, a photodiode and an electron analyzer.

As shown in Fig. 1 ▸, the setup has an FZP and an OSA collinear to the monochromated X-ray optical path. The sample is placed near the OSA and the photoelectrons are collected from the backside by the electron analyzer at an angle of 45°. A photodiode is placed in the X-ray optical path to obtain an image from the transmitted X-rays. Also, the diode allows acquiring maps of the X-ray absorption spectra both in transmission and in fluorescence yield. Overall, our instrument combines surface-sensitive spectromicroscopy (SPEM) with bulk-sensitive spectroscopy (STXM). It is a powerful and versatile tool capable of providing information about the chemical state of the bulk and interface at the same time, allowing a complete characterization of a sample under reaction conditions (*in situ* and *operando*). In a typical measurement with this setup, the photodiode and electron analyzer can record the signals independently and simultaneously. In the following sections the design and operation of the setup are given in detail.

## 2. Materials and methods   

The setup is a transportable high-vacuum chamber, which houses X-ray focusing elements, a sample holder and a photoelectron analyzer. The setup can be connected to the synchrotron beamlines through a 45° customized flange. The commissioning experiments were performed at the SIM beamline of the SLS at the Paul Scherrer Institute (Flechsig *et al.*, 2010 ▸).

### 2.1. X-ray source, SIM beamline   

The SIM beamline is a soft X-ray beamline providing tunable X-rays with photon energies in the range 90–2000 eV. It is equipped with two Apple II undulators (56 mm period length and 32 periods) followed by a plane-grating monochromator and employs a collimated light scheme to provide an intermediate focus for a permanently installed PEEM (Le Guyader *et al.*, 2012 ▸). By retracting the sample in PEEM and utilizing a second re-focusing mirror downstream, a near 1:1 image of the intermediate focus is available for the operation of a variety of exchangeable endstations (Flechsig *et al.*, 2010 ▸; Le Guyader *et al.*, 2012 ▸). During measurements, we used an unfocused beam to fully illuminate the FZP with the possibility of inserting a pinhole into the PEEM to obtain a well defined secondary source (Hovland, 1977 ▸). For the present experiments, we used horizontally, linearly polarized X-rays. The photon energy was set to 800 eV.

### 2.2. X-ray focusing elements and testing of the lateral resolution with a standardized sample   

**Figure 2:**
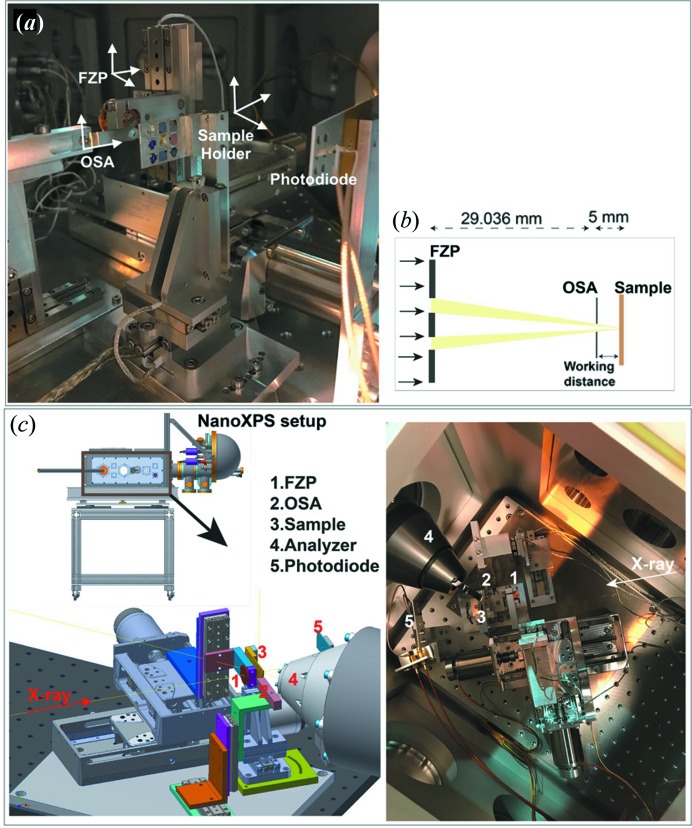
(*a*) X-ray focusing optics with motion of axes for each component, (*b*) schematics showing FZP and OSA with respect to the sample, (*c*) CAD-diagram and image of all the components in the assembly.

**Figure 3:**
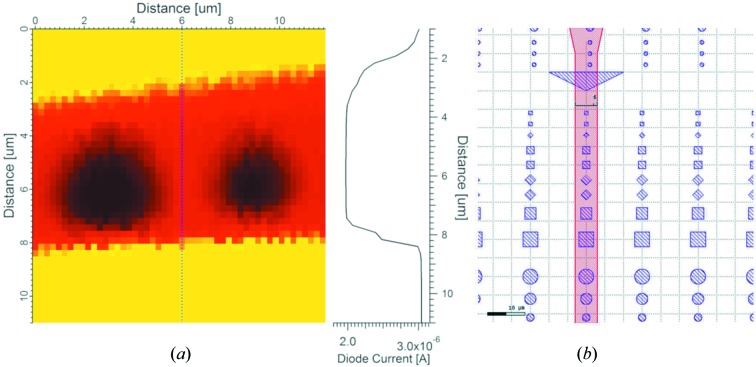
(*a*) Scanning transmission X-ray image of a copper stripe (6.0 µm width, red color scale) deposited on circular nickel microstructures (black color scale) on a silicon nitride membrane and the corresponding line profile (diode current acquired in transmission) across it. (*b*) Sketch of the copper stripe and of the nickel microstructures deposited on the silicon nitride membrane.

The use of diffractive X-ray optics like FZP for X-ray focusing is routine in many installations today, including the STXM setup at the PolLux beamline of SLS (Raabe *et al.*, 2008 ▸). We used a similar assembly here. Fig. 2 ▸ gives an overview of the setup. The FZP is placed in the optical path of the monochromatic X-ray and focuses the X-rays. Zone plates are circular diffraction gratings with a radially increasing line density. Like other diffraction gratings, zone plates are highly chromatic optics, and hence the focal length depends on the photon energy of the incident light (Günther, 2002 ▸). The photon-energy-dependent focal length of a zone plate *f*(*E*) is given by *f*(*E*) = *d*_FZP_Δ*r*(1/*ch*)*E*, where *d*_FZP_ is the diameter of the outer ring, Δ*r* is the size of the finest ring at the outermost zone, *c* is the speed of light, *h* is Planck’s constant and *E* is the photon energy. It means that for a zone plate of 900 µm, with the thinnest ring size of 50 nm and using a photon energy of 800 eV, the focal length would be 29.036 mm. The non-diffracted zero-order beam is rejected by using a central stop in the zone plate. Higher diffraction orders are sorted by using an OSA placed between the FZP and the sample. The OSA is placed near the focal point of the FZP to achieve a maximum rejection of the higher-order lights and mostly first-order lights pass through its aperture of 50 µm. The optimum working distance between the OSA and the sample can be determined by calculating the ratio between the OSA diameter and the FZP diameter multiplied by the focal length, *i.e.* the cone of the focal light has to be smaller than the diameter of the OSA at its position. The distance between the sample and OSA is kept constant and is called the working distance [Fig. 2 ▸(*b*)]. The FZP (*x*, *y*, *z*) and the OSA (*x*, *y*, *z* = constant, optical axis) are mounted on motorized and on piezonanoscanner stages, respectively, for alignment translations. The axes of movement for each component are shown in Fig. 2 ▸(*a*). During the alignment, the individual translations of the FZP, the OSA and their relative movements are controlled by software developed in-house for the operation of the STXM at the PolLux beamline. OSA alignment scans were performed to bring the OSA onto the optical axis defined by the zone plate and to calibrate the distance between the zone plate and the OSA. This was done prior to moving the sample into the beam by detecting the X-rays with the transmission diode. The alignment was checked a few times and was stable during beam time. For achieving the highest spatial resolution, a multi-channel plate detector might be used to check the alignment after every measurement in a faster and more reliable way. Fig. 3 ▸(*a*) shows a scanning transmission X-ray image (recording the beam current transmitted through the sample) of a ‘standardized’ sample, which is commonly used in STXM to test the lateral resolution of the instrument. Such a sample was lithographically patterned on top of a 100 nm-thick Si_3_N_4_ window. Nickel microstructures (of different shapes and dimensions) were deposited on top of the membrane and some of them were covered by a copper stripe having a width of approximately 6 µm [see Fig. 3 ▸(*b*)]. The line profile acquired at the edge of the strip line [Fig. 3 ▸(*a*)] shows that the lateral resolution is about 500 nm. Because all the samples were mounted on the same holder, the same focused beam was employed to obtain the XPS images shown in the proof-of-principle experiment (Section 3). The microstructures deposited on the ‘standardized sample’ were not grounded, and thus could not be probed by XPS. Therefore, a customized conductive sample (described in Section 2.3) was fabricated to carry out space-resolved XPS measurements in transmission mode.

### 2.3. Sample holder and sample   

The sample holder is a 30 mm × 20 mm and 0.5 mm-thick aluminium plate with nine equidistant round holes of 1 mm diameter. The samples were glued to the holes with an electrically conductive adhesive (silver ep­oxy). The plate was fixed on a stage equipped with three piezo positioners (Smaract GmbH) to move it in all three directions (*x*, *y*, *z*). The whole sample stage was again mounted on a fixed plate, and its relative angular position could be adjusted by moving it in a mechanical guide fixed with screws. The sample used in the present measurements was fabricated in order to be able to acquire photoelectron spectroscopy measurements. Since electrical conductivity is necessary to avoid charging of the sample during measurements, a 100 nm-thick silicon nitride membrane was covered with a continuous 5 nm-thick layer of chromium. Copper microstructures (thickness of approximately 15 nm) were subsequently lithographically patterned on top of the chromium layer. The chromium layer was deposited by means of thermal evaporation on the silicon frame of the membrane, and was essential to ground the samples to avoid charging/differential charging during SPEM measurements. A bilayer of methyl-methacrylate and of poly methyl-methacrylate was spincoated on top of the substrate and exposed with a 100 kV electron beam writer (Vistec EBPG 5000Plus), with a dose of 1500 µC cm^−2^. After e-beam exposure, the exposed resist was removed by immersing the sample in a solution of methyl-isobutyl-ketone and iso­propyl alcohol (1:3 in volume), followed by rinsing in pure iso­propyl alcohol. A layer of Cu (15 nm) was deposited by thermal evaporation in a Balzers BAE250 evaporator. The microstructures were then lifted off by immersing the sample in pure acetone. The silicon nitride window was then glued to one of the holes of the sample holders. The surface onto which the microstructures were deposited was opposite to the X-ray incidence.

### 2.4. Electron analyzer and data acquisition   

A Phoibos 150 electron analyzer equipped with a 1D delay line detector collected the photoelectrons from the backside of the sample. Such a detector was chosen specifically as it was developed for the normal detection and time-resolved detection of electrons, maximizing their transmission to obtain high-resolution XPS. The spectrometer is positioned at an angle of 45° with respect to the X-ray optical axis. It can operate both in fixed (snapshot) mode, thus setting the kinetic energy of the photoelectrons and acquiring a kinetic energy window having a specific width depending on the pass energy employed, and in swept mode.

The SLS beamline support for controls and data acquisition is integrated into the machine control system. It is based on *Experimental Physics and Industrial Control System* (EPICS) (https://epics.anl.gov), a client-server toolkit with Channel Access servers running on VME processors (PowerPC), and clients running Linux or NT PCs (Hunt, 1999 ▸). The native source code of the analyzer operation was modified to access it for remote operation. A simplified software running in EPICS was developed and could control the operation of the Phoibos spectrometer both in fixed and in swept mode by setting the analyzer pass energy and the kinetic energy range, respectively. Concerning the data acquisition software, we integrated the analyzer operation as process variables for the EPICS channel. The STXM software controlled the piezo stages, read back the data obtained by the analyser and wrote it to data files. The program allowed recording simultaneously the photoelectron signals and current measured at the photodiode with automatic raster, scanning the sample across the X-ray spot with nanometre precision.

## 3. Results and discussion   

### 3.1. Proof-of-principle experiments   

All experiments were performed using a 900 µm FZP and 50 µm aperture OSA. An 800 eV photon energy was used for probing the Cu 3*s* photoemission signal. At this experimental setup, the working distance between the OSA and the sample was fixed at 500 µm. The base pressure of the chamber was 1 × 10^−6^ mbar. Copper microstructures deposited onto a thin layer of chromium covering a silicon nitride membrane and its frame (as described in Section 2.3) were used as samples. A ‘high-magnification’ lens mode was used to avoid straying magnetic fields inside the chamber, as during the commissioning stage no mu-metal shields were installed. During the measurements, the sample was scanned in the *xy* plane (*z* is along the optical axis) at a step size of 500 nm by using piezopositioners (SmarAct, GmbH), while recording photoemission signals in a fixed mode and the current signals at the photodiode at each position. The minimum (theoretical) beam spot size achievable with the FZP used in this experiment, calculated using the Rayleigh criterion, was approximately 60 nm (1.22 nm × 50 nm, where 50 nm is the thinnest ring size of the zone plate). The estimated photon beam size used during this first commissioning was ≤500 nm, because the full unfocused beam (square, *ca*. 1.4 mm sides) was used to illuminate the FZP.

**Figure 4:**
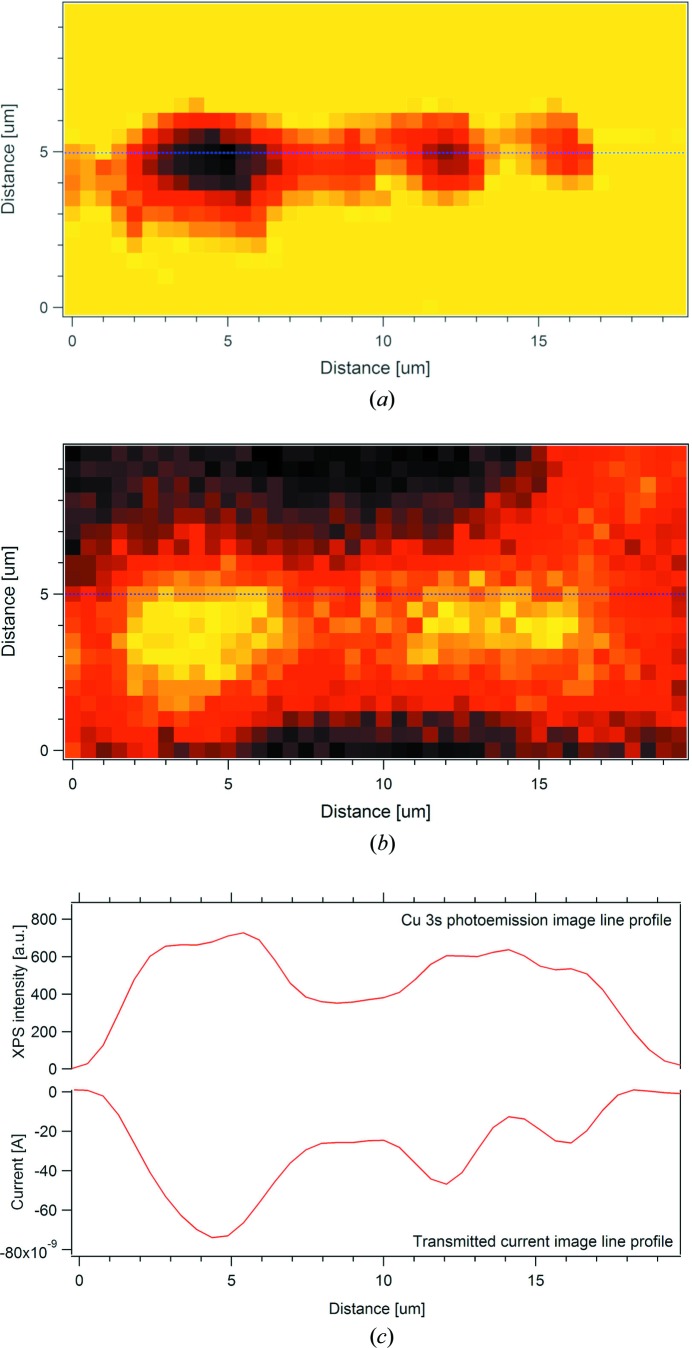
(*a*) Current image and (*b*) Cu 3*s* photoemission image of a 10 µm × 20 µm area of the sample; (*c*) horizontal line profiles for both images at the same position. Horizontal line profiles were smoothed three times using a binomial algorithm.

**Figure 5:**
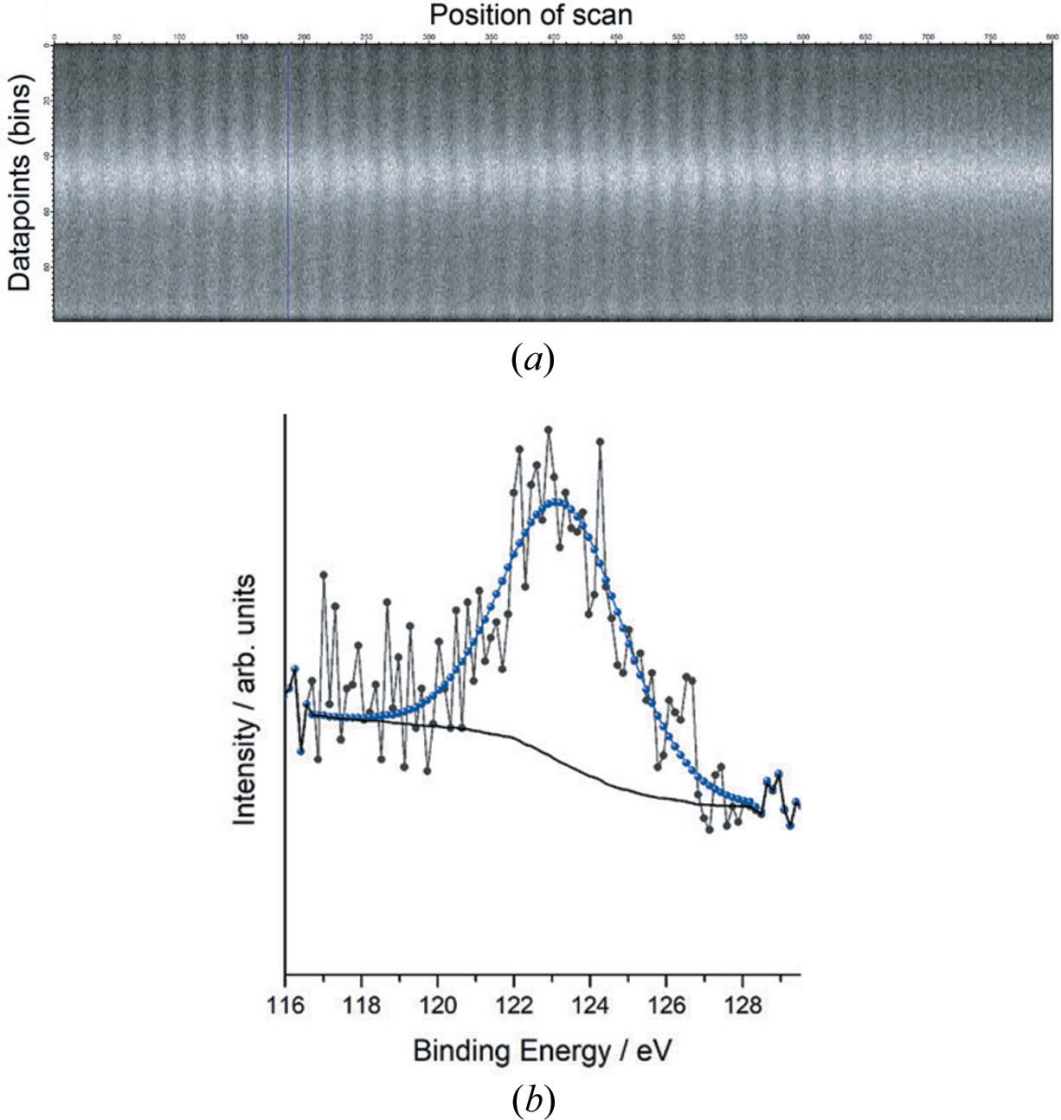
(*a*) Detailed XPS data of Cu 3*s* for the 10 µm × 20 µm area scan; (*b*) Cu 3*s* signal of the 187th line, *i.e.* from the (9,7) point.

Fig. 4 ▸(*a*) shows a transmitted current scan image of an area of 10 µm × 20 µm. The dark spots denote the location of the copper structures, having decreasing sizes estimated at 11 µm, 6 µm and 4 µm, respectively, from a horizontal line profile. The photoemission signal of Cu 3*s* was acquired in the same area of the sample reported. Although the Cu 3*p* photoemission signal has a larger photoionization cross section than the Cu 3*s* at the excitation energy used in the experiment, it is superimposed to the Cr 3*s* generated from the conductive chromium layer. The behavior of the integrated area underlying the Cu 3*s* signal is presented in Fig. 5 ▸(*b*). The copper-dense regions are where the particles are located. As expected, the maps of Figs. 4 ▸(*a*) and 4(*b*) show opposite color scales. The diode shows a minimum (dark-red color scale) where the copper structures are, because the sample is thicker and the beam is more attenuated, whereas the photoemission signal of Cu 3*s* shows a maximum (yellow–white color scale) in the same areas. Based on the diode image, the copper structures appear spatially separated, whereas the surface-sensitive XPS measurement suggests that copper spreads on the chromium support, ‘merging’ the particles together. The comparison between the horizontal line profiles acquired in the same position highlights the complementarity of the methods, as well as the potential of the instrument.

The kinetic energy of the electron analyzer was set to 122.5 eV. The selected pass energy of 100 eV allowed the acquisition of a kinetic energy window having a width of approximately 12 eV and a single snapshot was acquired for a dwell time of 1 s and summed ten times. The XPS signals were recorded in 100 energy steps (or bins) of the 1D delay line detector. Therefore, an XPS map is a matrix array containing 20 horizontal and 40 vertical points corresponding to a 10 µm × 20 µm area scan in a row as the *x* axis and where each point has 100 energy points read from the analyzer as the *y* axis. Fig. 5(*a*) ▸ shows the detailed Cu 3*s* XPS matrix where each vertical line represents an XPS signal corresponding to a specific pixel. By integrating each vertical line, corresponding spectra for each pixel of the sample can be plotted. For example, the XPS signal at the (*x*, *y*) position will be at the (*x* × 20 + *y*)th line. Fig. 5 ▸(*b*) shows the Cu 3*s* data of the (9,7) point, *i.e.* 187th line, which represents a copper structure. A binding energy of 123.3 eV for Cu 3*s* represents copper in cationic form. The full width at half-maximum is 3.76 eV. Based on this value and taking as a reference the natural line width of Cu 3*s* in a Cu(111) sample (Huff *et al.*, 1997 ▸), the overall energy resolution of the instrument can be estimated as 2.4 eV. This value can certainly be improved by acquiring the spectra in swept mode, *i.e.* decreasing the pass energy of the analyzer and pre-setting the acquisition time for each single energy point. Integrating the matrix array in different regions of the sample can help our understanding if the sample composition is homogeneous (as in our case) or if the same element is present in different oxidation states/compounds. Furthermore, acquiring the same SPEM map using increasing excitation energy allows probing of the sample at increasing depth, as the photoelectron kinetic energy of the same core level increases accordingly. This might give important information about the nature, depth profile and the time evolution of species involved in a reaction, and help to differentiate them from spectators (Artiglia *et al.*, 2017 ▸).

## 4. Conclusions and outlook   

We have presented a new instrument capable of simultaneously acquiring STXM and XPS two-dimensional maps of planar samples deposited on silicon nitride membranes with high spatial resolution. The instrument operates in transmission geometry, *i.e.* scanning a sample with a focused photon beam through a silicon nitride membrane while collecting both the transmitted photon current and the photo-emitted electrons. The setup has been tested at the SIM beamline of the SLS. First measurements with a model copper microstructure sample have successfully demonstrated the operation of the instrument. The results show element-specific photoemission spectra in transmission mode and an image from transmitted X-rays over a microscopic area with a resolution of ∼1 µm.

This setup offers the opportunity to combine surface-sensitive (photoelectron spectroscopy) measurements with bulk-sensitive ones (absorption spectroscopy), which can be acquired either in transmission or in fluorescence mode at the same time. Qualitative (element specific) and quantitative analyses can be carried out at different, tunable, investigation depths. In the next step, we aim to improve the lateral resolution of the setup. To improve the space resolution, optimization of parameters such as the size of the FZP, tight OSA-to-sample distance and X-ray flux on the sample will be crucial. Because our main goal is to test the transmission measurement geometry under ambient pressure conditions, *i.e.* coupled with a differentially pumped photoelectron analyzer, obtaining an ultimate resolution in the <100 nm range may be challenging. We expect that vibrations produced by the differential pumping stages (turbomolecular pumps) may be a limiting factor. To prevent such an issue, a mechanical damping of the beam-focusing element and of the sample stage can be foreseen and integrated in the design of the setup. The experiments shown in this manuscript were performed under high vacuum (10^−6^ mbar range). No modifications of the zone plate were observed during/after the measurements. The use of such a setup in an APXPS setup is still a perspective, and beam ionization of gas molecules in the millibar range might be a relevant issue affecting the performance of the setup and the lifetime of the ZP, which may need additional attention.

As a potential application, such a setup could be employed to scan ordered collections of metal nanoparticles deposited on selected substrates. A combined microscopic–spectroscopic analysis of the system may help to identify the interplay between metal and support, as recently demonstrated for hydrogen spillover by means of XPEEM (Karim *et al.*, 2017 ▸). The electron analyzer can operate up to 10^−4^ mbar, but an upgrade to the near ambient pressure version would allow experiments in the millibar range, thus partially filling the pressure gap.

Future developments include analysis of new and challenging samples such as dispersed-powder catalysts, and tailored oxide nanomaterials or model catalysts created by nanofabrication. The present version of the setup can operate up to 10^−4^ mbar, but can be upgraded to the millibar range in future. This will allow measurements of well characterized model samples under catalytically relevant conditions.

One of the aims of this setup will be moving toward experiments with membrane-covered *in situ* cells. The *in situ* cells that contain silicon nitride on one side and electron semi-transparent membranes on the opposite side shall be used. XPS measurements at 1 bar have already been obtained by means of these cells. Combining these small cells with the setup shown in this paper will make it possible to acquire spatially resolved spectroscopic images under catalytically relevant conditions.
